# Factors Influencing the Health Behaviour of Indigenous Australians: Perspectives from Support People

**DOI:** 10.1371/journal.pone.0142323

**Published:** 2015-11-24

**Authors:** Pippa Waterworth, Melanie Pescud, Rebecca Braham, James Dimmock, Michael Rosenberg

**Affiliations:** 1 School of Sport Science, Exercise and Health (M408), University of Western Australia, Crawley, Australia; 2 Regulatory Institutions Network, Australian National University, Canberra, Australia; Queensland University of Technology, AUSTRALIA

## Abstract

Disparities between the health of Indigenous and non-Indigenous populations continue to be prevalent within Australia. Research suggests that Indigenous people participate in health risk behaviour more often than their non-Indigenous counterparts, and that such behaviour has a substantial impact on health outcomes. Although this would indicate that reducing health risk behaviour may have positive effects on health outcomes, the factors that influence Indigenous health behaviour are still poorly understood. This study aimed to interview people who support Indigenous groups to gain an understanding of their views on the factors influencing health behaviour within Indigenous groups in Western Australia. Twenty nine people participated in the study. The emergent themes were mapped against the social ecological model. The results indicated that: (1) culture, social networks, history, racism, socioeconomic disadvantage, and the psychological distress associated with some of these factors interact to affect health behaviour in a complex manner; (2) the desire to retain cultural identity and distinctiveness may have both positive and negative influence on health risk behaviour; (3) strong social connections to family and kin that is intensified by cultural obligations, appears to affirm and disrupt positive health behaviour; (4) the separation between Indigenous and non-Indigenous social connection/networks that appeared to be fostered by marginalisation and racism may influence the effect of social networks on health behaviour; and (5) communication between Indigenous and non-Indigenous people may be interrupted by distrust between the groups, which reduces the influence of some non-Indigenous sources on the health behaviour of Indigenous people.

## Introduction

The disparities between the health of Indigenous and non-Indigenous populations continue to be prevalent within Australia [1–3]. Indigenous Australians have a shorter life expectancy (10.6 years less for males and 9.5 years less for females) and worse health outcomes than their non-Indigenous counterparts [1,2]. According to Vos et al. [4], 70% of the health gap between Indigenous and non-Indigenous Australians can be explained by non-communicable chronic diseases, with cardiovascular disease being the largest contributor (23%), followed by diabetes (12%), mental disorders (10%), and chronic respiratory disease (9%). It is recognised that health risk behaviour has a substantial impact on many non-communicable diseases [4–6], and Indigenous Australians more frequently engage in detrimental health risk behaviours, such as smoking tobacco and misuse of alcohol and drugs [4]. For example, in a recent national survey, 43.8% of Indigenous Australians were reported to be current daily smokers, compared with 15.7% of non-Indigenous Australians [7]. In light of the association between health risk behaviour and health outcomes, reducing health risk behaviour among Indigenous Australians may improve their health outcomes. Indeed, any efforts to curb health risk behaviours need to be considered within the broad social, economic, and political context in which individuals live and the way these social determinants of health impact daily living conditions and subsequent health risk behaviours [8–10]. From an equity perspective, it is necessary to critique the culture of the Australian public health system and note the ineffectiveness of many mainstream health interventions to effectively target Indigenous populations [11].

The factors that influence Indigenous Australians’ health behaviour are poorly understood [12]. Commonly, behaviour is believed to be driven by factors within the immediate control of the individual, such as perceived behavioural control and attitudes towards the behaviour [13]. However, research indicates that factors beyond the choice/control of the individual may influence Indigenous Australians’ health behaviour [14,15]. For instance, in some circumstances, the social disruption associated with historical events appears to promote health risk behaviours [16–19]. In particular, alcohol abuse and tobacco smoking have been linked with social disruptions [16], and the stress associated with daily life may exacerbate the practice of health risk behaviours such as tobacco smoking [14,19]. It has also been identified that social networks may promote health risk behaviours such as smoking [19]. Conversely, cultural identity and distinctiveness may be psychologically protective and therefore promote positive health behaviour [20–22].

During the colonisation of Australia, racism was pervasive [23]. Indigenous people were forced to live in missions and on reserves where freedom to hunt, socialise with relatives, practice traditional ceremonies, and marry were restricted [24]. Segregation and assimilation policies were introduced, along with institutionalisation and geographical restrictions [25]. In some instances, Indigenous people were prevented from speaking their traditional language, practicing their culture, and teaching their children their history and traditions [26]. The restrictions and conflict associated with colonisation led to loss of liberties and life and irrevocably altered Indigenous Australians’ cultural and social behaviour [27].

Past atrocities and continued discrimination have created long-term physical and psychological effects on Indigenous people that are often trans-generational [28]. This is reflected in numerous statistics and psychosocial circumstances that indicate that discrimination and racism towards Indigenous people still affects their lives [28–31]. For example, unemployment rates reveal a disparity, with 17% unemployment for Indigenous people compared with 5% for non-Indigenous [31]. Daily stressors related to such economic disparity also contribute to health disparities [32].

While this may indicate that the health behaviour of Indigenous Australians is affected by psychosocial circumstances beyond their control, there appears to be limited research exploring this aspect [12,33].

The association between health behaviour and factors that are beyond the control/choice of the individual suggests a broader psychosocial approach, employing a macro perspective, may be beneficial. This perspective enables the examination of factors that are associated with personal choice/control, in conjunction with factors that are beyond the choice/control of the individual [34,35]. Thus, factors that are traditionally considered instrumental in behaviour development could be considered simultaneously with factors that may be more influential within an Indigenous context [32,36,37]. Enquiry including sociological and psychological perspectives can be facilitated by the use of conceptual frameworks/models to assist the process of analysing the data [34,35]. One psychosocial model that has found traction within health research, including Indigenous health research [15,38–40], is the social ecological model [34,35]. Lynch’s [35] iteration of this model categorizes factors into a series of social levels that impact upon the issue under investigation. These levels include broader societal influences (i.e., culture, politics, and discrimination), neighbourhood or community social interactions (social interactions and connections through groups and organisations), close social connections (i.e., friends and family), and individual characteristics (i.e., behavioural and psychological). This model may assist when analysing data associated with understanding a diverse range of factors that affect a topic under investigation.

Insights from observers who are closely associated with a particular group or individual provide data from an opportune perspective that may enable new revelations regarding the factors that influence health behaviour, especially when analysed using a social ecological approach. In particular, people who provide support for groups or individuals are in a unique position to observe the people they support and the broader influences on the circumstances surrounding those people. Therefore, their perspective may encompass information regarding societal and community level factors, in conjunction with information regarding the effect of closer social connections. Prior research indicates that observers’/informants’ perspectives have provided valuable insight into the behavioural motivations of those they have personal or professional relationships with [14,41–44]. For example, Wood et al. [14] examined the factors that influenced Indigenous pregnant women’s smoking behaviour by seeking the opinions of health workers. Similarly, when Barnes et al.[41,45] sought to understand the factors that influenced Indigenous health behaviour, allied health professionals in support roles (i.e., nurses and health workers) were interviewed. Although the opinions of people in support roles have been included in some Indigenous health related studies, the type of roles often appear to be limited to health professionals. In order to gain a wider perspective, it is considered advantageous to include an interdisciplinary approach [46,47]. Consequently, the opportunity to include other support people such as educationalists or community development officers may provide a wider perspective on the factors that influence health behaviour. An integral aspect to such recruitment is the inclusion of Indigenous participants who, as well as providing insights relating to the people they support, are able to draw on their own experiences as Indigenous community members [14,44].

Attempting to further the understanding of Indigenous health behaviour by consulting a broad range of people who support Indigenous groups, but that also include Indigenous people, may have merit. Support workers are often exposed to the socio-political aspects of Indigenous health, as well as Indigenous people accessing community (i.e., health, education, or housing) services and as such are in an opportune position to provide a unique perspective on the situation under investigation [48]. They may also observe Indigenous community members attempting to moderate their health behaviours [14]. Wood et al. (2008) interviewed health workers during their investigation exploring the reasons why Aboriginal women continue to smoke during pregnancy. They included health workers because these support people were able to observe Aboriginal women during their pregnancy; at stage of when many people quit smoking. As a group, support workers can provide a useful perspective of Indigenous health across each of the main socio- ecological levels [38]. In light of prior research, the current qualitative study was designed to better understand the factors influencing health behaviour within Indigenous groups in Western Australia by mapping the emergent themes from discussions with people who support Indigenous groups into the social ecological model.

## Method

### Methodology

The methodology was guided by previous Indigenous research, including research conducted by Indigenous researchers and undertaken on Indigenous people, [14,38,41,48] and by an advisory group comprised of 14 people, including Western Australian academics and others involved in Indigenous health, including two Indigenous community members. The National Health and Medical Research Council (NHMRC) guidelines for ethical conduct of Indigenous research were adhered to throughout the planning, implementation, and analysis stages of this research. In accordance with these principles, the research team liaised with Indigenous health organisations within the State. Consequently, the research was designed to: respect the privacy and integrity of those who were involved in the interviews and the groups they supported; uphold the responsibility to convey the participants’ opinions accurately; aid reciprocity by providing an opportunity for people to share opinions with others in a non-judgemental setting; and ensure an open invitation to be involved in the research in order to promote inclusivity and equality.

### Recruitment

In an attempt to obtain a geographically diverse sample comprised of both Indigenous and non-Indigenous participants, the recruitment process included four aspects: (1) advertising the study on a website; (2) placing a recruitment booth at a health conference; (3) approaching relevant individuals; and (4) employing snowball recruitment techniques (as per [49]). Potential participants were screened to ensure they had close relationships with the Indigenous people they supported; details of the screening process are outlined below.

### Sample

In-depth interviews were conducted with 29 people (13 males and 16 females) who support Indigenous groups, and whose ages ranged from mid-twenties to late fifties. In order to examine possible perceptual differences between Indigenous and non-Indigenous support people, a similar distribution of both groups (Indigenous, n = 13; non-Indigenous, n = 16) was included in the sample. The support people involved were experts within their fields, which included education, community development, health promotion, counselling, community management, and health. The participants also had extensive experience (from four to 20 years) working within Indigenous communities. The majority of the support people had lived within the community they worked with for several years, enabling them to build strong relationships with community members. A few of them were in transitory roles, making regular trips to the community for a few weeks at a time. All the Indigenous support people lived and worked within their own communities, and had done so for many years. All participants held current positions where they were employed to provide support to Indigenous community members. Seven participants were involved with metropolitan groups, six were involved with regional groups, six with remote groups, and 10 were involved with a combination of geographical localities. This reflected the distribution of the Indigenous population within the State, where 40% of the Indigenous people live in remote/very remote locations, 37% live in urban settings, and 13% live in regional locations [50]. All participants had strong networks within the communities they supported. These networks included both Indigenous and non-Indigenous people, and in most cases the networks comprised formal and informal relationships. This breadth of knowledge and experience meant the participants were able to provide insights from a unique perspective.

### Procedure

Ethics approval for this research was obtained from the Western Australian Aboriginal Health Ethics Committee and The University of Western Australia Human Research Ethics Committee. All participants provided their informed written consent to take part in the study prior to the commencement of data collection. Interviews took approximately one hour and were conducted at venues convenient to the participants. The interviews involved open-ended questions from a semi-structured interview guide designed to encourage the respondents to discuss their thoughts regarding factors that influence Indigenous health behaviour. In order to build rapport, the questioning began by asking the participants about their background. This was followed by a discussion centred upon the key issues affecting the people they interact with and how these issues affect their health behaviour. The questioning was iterative, allowing participants to expand on areas they felt were most important. During the data collection, the lead researcher also made field notes.

### Analysis

All the interviews were audio recorded and transcribed verbatim. Transcripts were uploaded into NVivo10 (QSR International Pty Ltd., Melbourne, Australia). The analysis commenced with the lead author, repeatedly reading the transcripts in order to immerse herself in the data, this was completed using an empathetic understanding, which has been identified as contributing to the trustworthiness of analysis [51]. All data were coded by the lead author and the data were analysed thematically via deductive and inductive processes [51,52] while using a constant comparison process to ensure analytical rigour [53]. The initial coding schema was developed using concepts from the social ecological model (as per [35]). The coding schema was updated regularly to incorporate emergent themes (as per [51]) and, when necessary, previously coded data were recoded to these new themes (as per [53]). The data were also subjected to thematic analysis using matrix searches [54]. The interpretations were compared with the lead researcher’s notes as a means of triangulation. These notes were also an avenue of reflection regarding possible bias; the notes and the subsequent reflexive process contributed to methodological and interpretive rigour (as per [55]). The authors, all non-Indigenous, participated in the discussions relating to the themes; this further contributed to the trustworthiness of the data by providing the opportunity to explore different perspectives. In addition, the potential for misinterpreting the data was minimised by an iterative, constant comparison method, combined with member checking and advice from the advisory group and a forum of experts, who were experienced professionals within health promotion (as per, [52,56])

## Results

**Fig 1:**
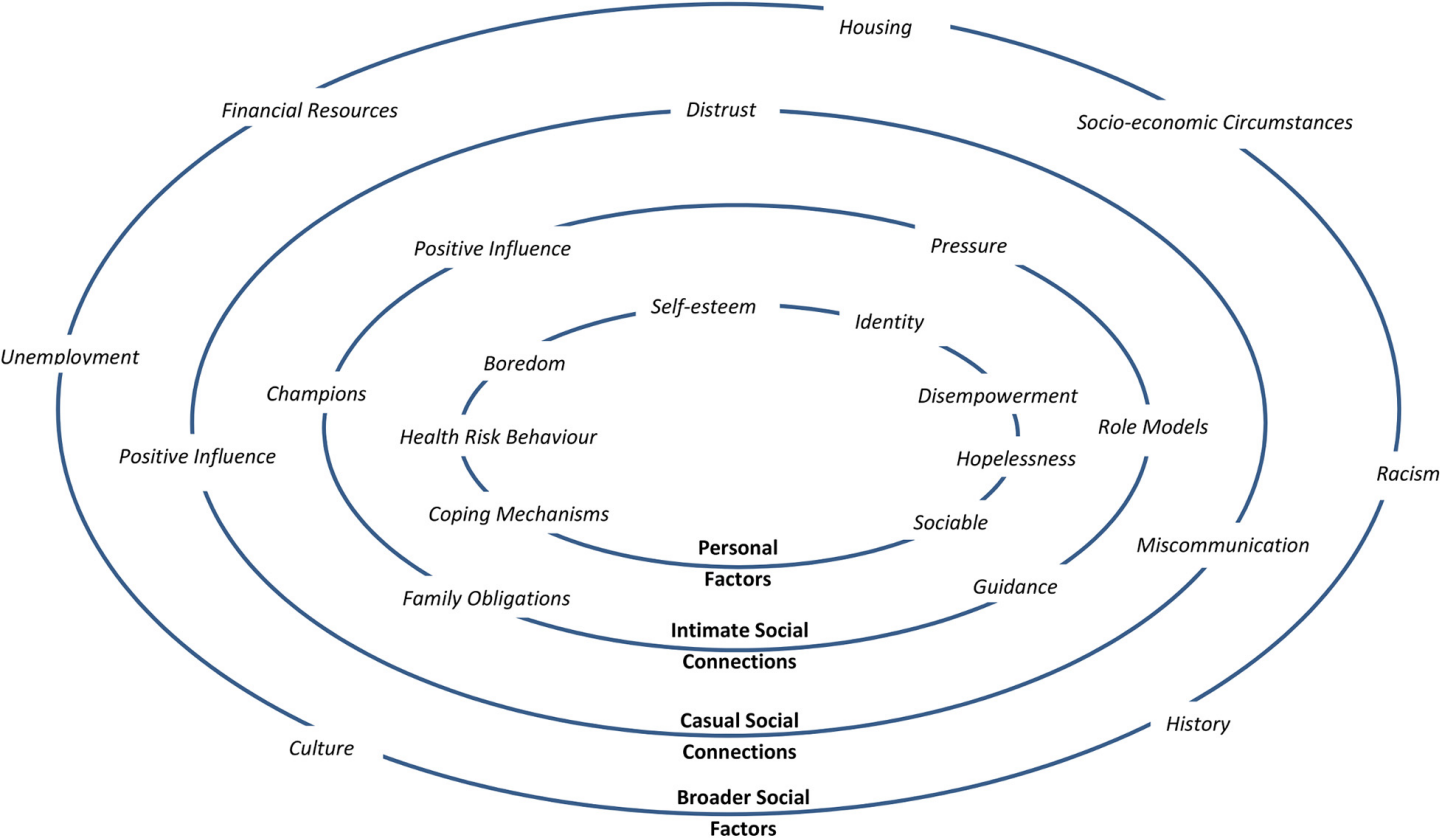
Factors influencing the health behaviour of Indigenous Australians from the perspective of people who support Indigenous groups.

The focus of the discussions was not limited to one type of health behaviour; the participants mentioned a range of health behaviours throughout the discussions. These included smoking, alcohol consumption, physical activity, and dietary habits. The themes identified in this study were analysed and categorised within a social ecological framework. Fig 1 provides a concept map of the findings presented in this format. Broader social factors relating to culture, history, racism, and socio-economic circumstances were thought to influence health behaviour. Social connections with a variety of Indigenous people including family, other Indigenous community members, and non-Indigenous people (including health professionals) were also thought to influence health behaviour. In addition, personal circumstances and psychological factors were considered influential. Many of the factors were understood to act in a complex manner with respect to their effect on health behaviour. No differences between Indigenous and non-Indigenous interviewees were identified, indicating alignment between the two culturally different perspectives with respect to the topic under investigation.

Study participants felt there were multiple factors that influenced the health behaviour of Indigenous people within the Western Australian communities they supported. The factors that emerged from conversations regarding remote, regional, and metropolitan Indigenous community members were the same. The similarity in factors identified at a variety of geographical locations may indicate these factors transcend other geographical concerns because of the magnitude of their effect upon the circumstances. The findings are presented under emergent themes that were identified during the data collection episodes.

### The perceived influence of socio-cultural factors on health behaviour

#### Culture

Culture was identified as fundamental to an Indigenous outlook on life, and therefore, substantially influenced health behaviour and factors that affect health behaviour. In particular, the cultural importance of connections within an extended family network, or kinship group, was emphasised as influential on health behaviour.

*It’s kinship*. *It’s sharing*. *They put their families first; it’s cultural*. (P15: Indigenous male, metro)

There was a very strong cultural obligation towards family, which includes providing support for family members. It was considered a cultural expectation to provide family (kin) with food, accommodation, or money, regardless of one’s own circumstances. Many participants (both Indigenous and non-Indigenous) commented that this cultural obligation to family impacted upon health behaviour. This occurred because they were often supporting a large network of people (as a result of the kinship system), thereby placing psychological and physical demands upon individual resources.

*A lot of obligation to their family…aunty or grandma or a child and his or her partner and their kids and whoever else is transient at that time that’s come to stay*. (P4: Indigenous male, remote)

*The family arrive or someone comes to stay and you’re obligated to feed them*, *you can just go and get fish and chips or some other fast food; it’s easy and cheap*. (P3: non-Indigenous male, regional/remote)

*Getting a job and earning money that goes into the individual’s bank account but it’s not really their money*. *They’ve got to share it out with whomever*, *if there’s some sort of relationship*. (P17: Indigenous female, metro/regional)

*Indigenous people won’t cook two different meals*, *you know; if someone in the family is a diabetic… they’ll eat whatever’s going*. (P18: Indigenous female, regional)

Participants mentioned that the Indigenous people they supported generally had a sociable, sharing lifestyle. In the context of economic hardship, the desire to share and be sociable encouraged acts such as sharing cigarettes, which were relatively cheap in comparison to cost of a meal, and could be shared without too much financial outlay. Participants also noted that smoking together produced a sense of camaraderie amongst the Indigenous people. These aspects reflect the influence of the Indigenous cultural perspective regarding sharing and being social:

*Quite often when you haven’t got a lot to offer other people or to share with other people*, *cigarettes are quite a big thing to be able to share with family or friends when they come and talk with you*. *So it’s almost like a cultural thing that we’re up against as well*, *that it’s not just a health behaviour issue*. (P24: non-Indigenous male, regional/remote)

Interactions with family and the kinship network were considered to have substantial influence upon individuals’ health behaviour. Respected individuals and elders acted as role models within the community, providing examples of and support for healthy behaviour. Conversely, it was observed that family and friends exerted pressure that led to unhealthy behaviours:

*Old ladies*, *old men and elders*, *they’ve got great influence [on others’ health behaviour]*. (P9: Indigenous male, regional)

*Respected people such as parents and elders are great roles models; they can they can positively influence Indigenous community members’ health behaviour*. (P3: non-Indigenous male, regional/remote)

*Peer pressure especially for the young guys*. *This influences people to do things they may not want to do*, *such as drinking alcohol*. *It’s happened to me*. (P4: Indigenous male, remote)

In light of the substantial obligations to family and community and their impact upon individuals’ attitudes and resources, the participants thought that it may be difficult for a single individual to change their health behaviour without the wider community implementing similar changes. One participant referred to this via the following analogy:

*Well*, *you put a whole lot of crabs in a bucket and one of them starts to claw its way out to the big outside world*. *The ones down below grab a hold of that one*, *and instead of them all climbing out*, *the first one gets dragged back in*. *They’re always trying to get there*, *but they get dragged back in*. (P24: non-Indigenous male, regional/remote)

#### Cultural preservation

Cultural identity had a unifying affect that led to an expectation to conform. In light of this, Indigenous people were sometimes criticised by others from their community, as a result of participating in lifestyle choices that were considered culturally inappropriate or non-Indigenous:

*If an Aboriginal person is doing really well*, *and they’ve got their own home and a car and a settled life*, *then they say [Indigenous people] you become less Aboriginal*. (P20: Indigenous female, regional)

Such comments reflect the tension that is created by attempting to accommodate two contradictory motivational drivers: (1) the desire for cultural identity and inclusivity; and (2) the influence generated from a source outside their cultural perspective.

There was a reluctance to conform to anything that was perceived as not culturally appropriate or that diminished one’s cultural identity. This influenced health behaviour, promoting behaviours that were considered culturally appropriate and, conversely, discouraging behaviours that were considered culturally inappropriate:

*When she was offered the choice between a white roll and a wholemeal one*, *she took the white one*, *pointing to the wholemeal one and saying “I don’t want that*, *that’s white fella food”*. (P29: non-Indigenous female, metro)

Support workers also mentioned that loss of identity, disempowerment, and a sense of hopelessness that was associated with the struggle to maintain their cultural identity encouraged Indigenous people to engage in unhealthy coping mechanisms.

*I think there’s a real lack of [cultural] identity*, *low self-esteem*, *and lack of the future*. (P6: non-Indigenous female, Metro)

*[Indigenous] people are so disempowered that they don’t even connect*, *“OK I can do that*, *I can make this [health behaviour] better”*. (P8: Indigenous female, all regions)

In unpacking the notion of cultural preservation, an interesting contradiction was apparent whereby there appeared to be a dichotomy of cultures occurring simultaneously. It was interpreted that culture could be both reinforcing of healthy behaviours and counterproductive. This once again highlights the nuanced and complex nature of Indigenous culture.

### The psychological influence of history and racism on social relationships on health behaviour

According to many participants, the traumatic history experienced by Indigenous Western Australians involving colonisation and oppression continued to have a psychological and physical impact upon them. The psychological affect caused by hearing the history and seeing the places where events occurred perpetuated the impact of the past. This was thought to produce psychological distress that impeded consideration of appropriate health behaviour; created psychological barriers between the Indigenous and non-Indigenous people; and increased the necessity for coping strategies.

*We were considered animals until they gave us the vote in ‘67*. *We were treated badly—I still pass the place where they massacred my family*. *People find that sort of thing hard to forget*. (P27: Indigenous male, regional)

Numerous references were made to continued racism towards Indigenous people within Western Australia. The constant presence of systematic racism, on a systemic, as well as interpersonal level, was believed to influence decisions regarding health behaviour. Racism was sometimes a barrier to accessing healthy choices or health education. In addition, exposure to suspicion produced psychological distress and avoidance behaviour.

*It doesn’t matter*, *a train station*, *just any shop you go into*, *if you’re black you get watched*, *it’s as simple as that*. *It’s horrible*. (P21: Indigenous female, metro)

*The nurse made a comment about not trusting them [an Indigenous family] while they stood next to the bed and could easily hear what she was saying*. *I could tell this upset them and made them wary of the nurse* (P22: non-Indigenous female, metro)

*They get excluded from information regarding health behaviour because of discrimination*. (P1: non-Indigenous, all regions)

On occasion, unhealthy options were made very accessible, in an attempt to convince community members to leave premises.

*The way to control things was they would give the Aboriginals free take-away food as long as they went away and ate it*. (P7: non-Indigenous female, regional)

In addition, health risk behaviours, such as smoking and consumption of alcohol and foods high in carbohydrates and fats, were introduced into the normative patterns of individuals and families.

*She said*, *“All these dependencies have been put on us*. *And then it’s monkey see*, *monkey do*. *My mother didn’t get money when she was working*. *She used to get flour*, *sugar*, *tobacco*.*”* (P10: Indigenous female, remote)

### The perceived influence of socio-economic circumstances on health behaviour

Socio-economic circumstances including economic hardship and unemployment diminished the financial resources available to fund health needs. It was felt that these financially challenging circumstances were also psychologically distressing, which compounded their influence on health behaviour. The outcomes of these circumstances were perceived to be overwhelming and prevented health behaviour from being prioritized. Overcrowded and inadequate housing were identified as common barriers to positive health behaviour. Living in overcrowded accommodation was thought to compromise many aspects of health behaviour, including nutrition:

*You’ve got ten people living in your house and we’re saying now you know you really need to eat some healthy tucker*. (P2: non-Indigenous female, regional/remote)

The psychological distress created by economic hardship reduced the capacity to focus upon health behaviour and increased the propensity to engage in unhealthy coping mechanisms such as smoking and alcohol use.

*It’s a really big thing with health behaviour because of the overcrowding in houses*. *I mean I know of a family where there are about 12 of them in a two bedroom unit*. (P26: Indigenous female, regional)

*You want to come back home and see where they live*. *God*, *I’d be drinking*, *every day too*. (P20: Indigenous female, regional)

It was acknowledged that engaging in health risk behaviours had a detrimental effect on Indigenous health outcomes. However, there was speculation that these behaviours were partially in response to the psychological distress caused by their circumstances. This is true, especially with respect to the use of smoking and drinking alcohol, which were used as coping mechanisms.

*The stress of all those social issues*, *the overcrowding in housing*, *unemployment*, *suicide*, *drug and alcohol consumption*, *all those sorts of things*, *means sometimes smoking is a comfort to people and their only means of actually having something*. (P3: non-Indigenous male, regional/remote)

### The perceived influence of social connections with people from outside the family/kinship group on health behaviour

It was often mentioned that interactions between Indigenous people and non-Indigenous representatives from organisations, especially health-related organisations, had the potential to positively influence health behaviour. These interactions provided practical support, encouragement, and information that promoted healthy behaviours. However, miscommunication was thought to reduce the positive influence that could be gained from these sources. Due to the role such institutions have played in controlling and regulating the lives of Indigenous Australians over time, Indigenous people may be wary of representatives of non-Indigenous organisations. This situation was exacerbated by people distrusting non-Indigenous representatives who were not familiar to them. It was also explained that people felt that organisations were not listening to them or criticised them, which produced a barrier to productive communication.

*They are often wary of white fellas and if they don’t know them they don’t really trust them*. *It’s understandable*, *given the history and the bad treatment that continues*. (P8: non-Indigenous female, all regions)

*They’re not going to start trusting a government organisation*, *when they’ve lost trust in them because of historical events*. *So they’re going to be less likely to listen to a [health] message if it comes from the government*, *than if it comes from Aboriginal people*. (P9: Indigenous male, regional)

*We’d spent literally months on these being culturally appropriate and sensitive pamphlets with images of Aboriginal people and terminology*, *same sort of thing*. *“Hey you mob*, *get deadly” [In an Indigenous context this colloquialism implies enthusiasm*, *encouragement or appreciation]*, *and they were of no interest to these people whatsoever*. (P2: non-Indigenous female, regional/remote)

Several participants’ comments indicated that people within the communities they supported resented being told how to live their lives or being criticised for not living in a certain manner. This resentment appeared to stem from the controlling or paternalistic sentiment that was related to being told how to live one’s life. Such comments highlight the challenges involved in providing health behaviour guidance in a manner that will engage community members and not exacerbate or reflect historical marginalisation:

*A lot of Aboriginal people are sick of being told how to live their lives and being criticised for how they live their lives and they want to hold onto the things that are important to them*. (P14: non-Indigenous female, metro)

## Discussion

This study explored Indigenous health behaviour in Western Australia from the perspective of support workers; around half of whom were Indigenous. The results reveal the complex relationship between social relationships and the broader Indigenous and non-Indigenous cultures and their impact on individual health behaviours. The results show that the participants in this study considered that the health behaviours of the Indigenous people they supported were extensively influenced by factors beyond the control of the individual. Culture, social networks, history, racism, socioeconomic disadvantage, and the psychological distress associated with some of these factors were thought to affect the health behaviour of the Indigenous community members supported by the participants involved in this study. In addition, the views expressed by all participants, regardless of their status as Indigenous or non-Indigenous were in alignment, with no differences observed.

It was apparent that these factors were often interrelated and, therefore, affect health behaviours in a complex manner that is not easily described. For example, inadequate housing leading to overcrowding may detract from an individual’s ability to enact positive health behaviours [20,21] However, the social and emotional wellbeing involved in interacting with other Indigenous people may negate or in some cases confound the effect of the overcrowding and have positive effects on health behaviour [20,21]. The intimate social connections that occur in such situations may have positive effects on psychological factors such as self-esteem, which can have a positive on influence health behaviour [20,21]. Further, it was apparent that the Indigenous culture was a strongly empowering and capacity building force within Indigenous communities. However, the unrelenting discrimination within the broader Australian community appeared to act as a moderating force that potentially undermined the empowering effects of the culture. The synergetic effect of such interactions impacted upon health behaviour.

Support workers’ accounts of the challenges of changing health behaviour focused on the importance of socio-cultural factors. This was primarily facilitated by the emphasis that was placed on social connections /networks by the culture. It was believed that some cultural aspects may have reinforced social connections in a manner that promoted group conformity and group expectations. This led to a complex interaction between socio-cultural factors and health behaviour. Consequently, it appeared Indigenous people were more likely to listen to advice which was provided by their relatives or other Indigenous people. Such reliance on advice from familiar sources is common in situations where cultures live together but have different socio-cultural traditions [57,58]. Moreover, the importance of relationships with family and kin, and the associated obligation to these relationships, significantly influenced health behaviour. In light of economic circumstances, one aspect that appeared to be particularly important was the cultural obligation to share resources with family members. Interviewees felt that this issue resulted in diminished resources available for maintaining personal health behaviour. Johnston and Thomas [59] noted that such cultural obligations add another layer of complexity to social dynamics that is not experienced by non-Indigenous groups. Passey et al. [19] also noted the importance of cultural identity (Aboriginal identity) in social network formation, and the role social networks play in perpetuating smoking behaviour. Similar to this, the current study highlights that in some instances, the desire (or perhaps obligation) to share that arises from, and reinforces, cultural identity may reduce the opportunity and inclination to make health behaviour adjustments, which are necessary for specific health issues, such as diabetes. This cultural focus on family and kin altered the priority of personal needs and the motivational influence derived from identity, self-esteem, and empowerment.

According to support workers, the importance of maintaining cultural distinctiveness impacted on the health behaviour of Indigenous people. Moreover, the continual experience of racism and discrimination may polarise Indigenous people towards their own culture, in a manner similar to that theorised in social identity theories such as in-group (us)/out-group (them) theory [60]. This theory states that people may develop strong links with a group they identify with and, conversely, can develop prejudice against opposing groups [60,61]. This may indicate that racism and discrimination have a confounding effect on cultural identity. In light of this, the necessity to maintain cultural distinctiveness may outweigh caution regarding the negative consequences of health risk behaviour. In their review of social theories, Dixon and Banwell [62] supports the notion that health risk behaviour is sometimes perpetuated in disadvantaged groups by the need to maintain distinctiveness from other groups. In a similar manner, a study involving Indigenous Australians from four rural communities within New South Wales indicated the necessity for cultural distinction may influence smoking behaviour [19]. Brough et al. [20] also suggested that cultural identity and cultural distinctiveness have important psychological protective qualities in the face of marginalisation and racism.

The importance of cultural distinction may promote resistance to embracing health behaviours that are perceived as being aligned to another culture which in turn could promote engagement with health risk behaviours or healthful behaviours depending on the situation. A social resistance framework has been devised in an attempt to explain such resistance within non-dominant minority groups [63,64]. In this context, engaging in health risk behaviours could be explained as everyday acts of resistance against the ideations and concepts of the dominant group [63]. This framework also explains minority group members’ criticism towards embracing attitudes and beliefs of the dominant group [63,65,66]. Factor et al. [64,65] used this framework to provide insight into health risk behaviour within the African American minority group in the United States. Given the sentiment expressed in the current study’s discussion, if positive health behaviours were perceived to be part of the non-Indigenous culture this may create a barrier to those behaviours.

The separation between Indigenous and non-Indigenous social connection/networks that appears to be fostered by marginalisation and racism may influence the effect of social networks. Support workers’ comments indicated that social connections with non-Indigenous people may be hindered by the maintenance of cultural distinctiveness, an oppressive history, and racism. This supports suggestions that marginalisation and racism impact upon social networks to diminish the availability of non-Indigenous connections [19,20,67]. Acceptance of health behaviour information that is derived from non-Indigenous sources or depicted from a non-Indigenous perspective may be diminished by the influence of marginalisation, racism, and desire for cultural distinctiveness.

Communication between Indigenous people and non-Indigenous people may also be disrupted by the sense of wariness or distrust some Indigenous people feel towards non-Indigenous people. The participants in the current study noted that distressing historical encounters and present day racism lead to an inclination by Indigenous people to distrust non-Indigenous people. Moreover, Indigenous participants’ comments indicated that the emotional distress and sorrow associated with tragic historical events, such as family massacres, impacted greatly upon Indigenous people. The detrimental impact of racism and historical events has been noted in previous studies that indicate such encounters have been psychologically distressing for Indigenous Australians [68,69]. Bond et al. (2012) noted that cognitive disparity may arise in response to advice regarding healthy living when provided by state institutions that were instrumental in the oppressive colonisation of Indigenous people.

Communication has a relational component that can be disrupted by distrust [70]. Gilson (2003) suggested that trust is affected by past encounters, either between people or people and organisations. Within an Australian Indigenous context, cultural emphasis on relationships and connections indicates that the importance of this relational aspect of communication may be heightened. Therefore, the trustworthiness of the messenger is a very important part of validating the information they convey. When the messenger is a stranger, trust stems from the reputation of the organisation they represent, or the credibility of known associates [70]. In light of this, preconceived distrust may have the potential to diminish the effect of communication from non-Indigenous sources. The findings in this study suggest that distrust may create a communication barrier with regards to promoting health behaviour to Indigenous Australians.

It was posited that it may be difficult for individuals to change their health behaviour without the wider community implementing similar changes. Research into the factors influencing health promotion within Samoan communities emphasised the conundrum that is caused when health is considered from an individually oriented perspective and from a culturally oriented social wellbeing perspective [71]. They concluded that this resulted in changes in health behaviour being suspended until cultural change occurs; thereby inadvertently placing the responsibility for improving health back on the cultural leadership. The notion that the normative behaviour of a group (for instance, a cultural group) will influence the individuals within the group is commonly acknowledged [72]. Such influence can encourage and spread behavioural change through the group’s social networks [13,72]. However, in instances where there is discrimination or oppression, the expectation for cultural leadership to champion health change may inadvertently exacerbate inequities. For instance, Hardin [71] suggested that when culture is acknowledged as the most important factor to changing health behaviour, health practitioners inadvertently reproduce structural inequalities in their encounters with clients. A re-framing needs to occur whereby there is much more importance and validation of Indigenous culture expressed through mainstream channels to cultivate a deeper population-wide understanding of the rich culture of Indigenous Australians.

## Limitations

Due to the qualitative nature of the study, the findings cannot be generalised beyond the study sample. However the credibility and validity of the themes was substantiated by the methodological and interpretive rigour employed throughout the study (as per [55,73]). The use of the social ecological model may have limited the scope of the findings. This model facilitated a broad inspection of the data, which may have detracted from examining minute differences in the data. However, in using this model the findings highlight factors that transcend the heterogeneity within Indigenous groups. In terms of methodological limitations, it is acknowledged that despite no differences being observed between the Indigenous and non-Indigenous informants, the recruitment of non-Indigenous people in support roles may be insufficient to explain Indigenous individual health behaviours. While Indigenous perspectives were obtained in the current study, future research should prioritise the perspectives of a larger and more geographically diverse sample comprised of only Indigenous people in explaining their own health behaviours to complement the findings of the present study. Further, in the current study, culture is portrayed as a homogenous entity, when in fact it is a nuanced and complex part of Indigenous populations’ lives that cannot be explicated fully from a small sample comprising both non-Indigenous and Indigenous informants.

## Conclusion

The current study highlights the complexities and challenges created by the intersection of (in broad terms) two cultures (Indigenous and non-Indigenous cultures). This dynamic is confounded by the oppressive history combined with racism that persists into contemporary circumstances, and the associated marginalisation. In addition to marginalisation, the desire for cultural distinctiveness and the influences of cultural perspectives further confounds and exacerbates the dynamics caused by other enablers and barriers, such as social connections and social support. In particular, the data suggests that distrust created by historical and contemporary racism may impede any health and broader assistance that might be gained from non-Indigenous sources. Conversely, it also highlights the conundrum involved in promoting health behaviour without reinforcing oppression or inequalities. It also points to the necessity for Indigenous community coalition driven efforts to improve health behaviour as a way of reducing inequities and empowering communities [74].
